# When Do Undergraduate Students Prefer AI? Insights into AI Scoring and Feedback

**DOI:** 10.3390/bs16071196

**Published:** 2026-07-15

**Authors:** Seyma N. Yildirim-Erbasli, Munevver Ilgun Dibek, Mackenzie L. Thomas, Nicolya Lesoway

**Affiliations:** 1 Department of Psychology, Concordia University of Edmonton, Edmonton, AB T5B 4E4, Canada; mlthomas@student.concordia.ab.ca (M.L.T.); nlesoway@student.concordia.ab.ca (N.L.); 2 Department of Educational Sciences, TED University, Ankara 06420, Turkey; munevver.ilgun@tedu.edu.tr

**Keywords:** artificial intelligence, student preferences, automated essay scoring, feedback, ChatGPT

## Abstract

The integration of artificial intelligence (AI) into higher education assessment has prompted growing interest in how students perceive and prefer AI involvement in scoring and feedback. While prior research has largely focused on technical performance and accuracy, this study aims to fill a gap in the literature by examining students’ preferences and perceptions regarding AI scoring and feedback, with particular attention to context, assignment stakes, and post-evaluation reflections. Ninety-three undergraduate students completed a survey consisting of Likert-type items, scenario-based questions, and an activity in which they generated AI-based scoring and feedback using ChatGPT. Results showed that students preferred structured, moderately detailed AI feedback, particularly for grammar and organization, but generally favoured human evaluation, especially for subjective tasks. While AI was seen as useful in lower-stakes contexts, concerns remained about its ability to assess more complex aspects of writing. Participants expressed a strong preference for hybrid approaches in which AI augments rather than replaces human judgment, along with a need for transparency and opportunities for human review. Collectively, these findings highlight that undergraduate students’ preferences are highly context-sensitive and role-specific, underscoring the importance of student-centred implementation strategies of AI in higher education.

## 1. Introduction

Artificial intelligence (AI) has become increasingly integrated into the core functions of higher education, influencing how universities teach, assess, and support learners (Hahn et al., 2021; Hockly, 2019; Lee et al., 2023). Recent advances in generative AI have enabled automated systems capable of evaluating student work and providing feedback at scale, now deployed across essay scoring, short-answer evaluation, and formative feedback platforms embedded within digital learning environments (Nazaretsky et al., 2025; Slepankova et al., 2025). These technologies offer tangible advantages: rapid response times, scoring consistency, and the capacity to support large-scale evaluation processes that would otherwise demand substantial human resources (Buzick et al., 2016; Kumar & Boulanger, 2021).

Yet the integration of AI into assessment practice raises questions that extend well beyond technical performance. Assessment is not a purely algorithmic procedure; it carries interpersonal, institutional, and ethical dimensions that shape how evaluation is perceived and accepted by learners (Er et al., 2025; Nazaretsky et al., 2025). Feedback supports learning only when students meaningfully interpret and actively use it (Lipnevich et al., 2016). The effectiveness of AI-supported assessment, therefore, cannot be reduced to scoring accuracy alone—it depends critically on how learners perceive these systems and whether they are willing to engage with them.

Three interrelated gaps in the existing literature motivate the present study. First, research has concentrated disproportionately on the technical performance of automated scoring systems, demonstrating that AI can achieve reliability comparable to human raters (Buzick et al., 2016; Flodén, 2025), while offering limited insight into how students evaluate these systems as participants in assessment. This omission is important because evidence from adjacent domains consistently shows that individuals may reject algorithmically derived judgments even when algorithms outperform human decision makers, a phenomenon documented in medical decision-making, hiring, and financial advising (Dietvorst et al., 2015; Gaube et al., 2021). Whether similar patterns characterize student responses to AI scoring and feedback in higher education remains underexplored.

Second, the literature often fails to distinguish between two conceptually distinct constructs. Perceptions refer to students’ evaluative beliefs about the usefulness, fairness, or credibility of AI systems, whereas preferences concern how students believe AI should be incorporated into assessment processes (Kasneci et al., 2023). Positive perceptions do not necessarily translate into preferences for AI involvement in assessment. Students may recognize the efficiency of AI while still preferring human graders for high-stakes evaluations, or they may hold nuanced views regarding the appropriate division of responsibilities between AI tools and instructors. Existing studies rarely examine these preference structures explicitly. Consequently, little is known about which feedback characteristics, such as tone, length, format, timing, and focus, students consider pedagogically valuable in AI-generated feedback, or how they believe AI and human instructors should divide assessment responsibilities (Le et al., 2025; Tossell et al., 2024).

Third, most empirical work relies on hypothetical scenarios in which students are asked to imagine how they would respond to an AI-generated assessment without directly experiencing it (Nazaretsky et al., 2025; Siregar et al., 2024). As AI tools become increasingly embedded in real learning environments, students’ perceptions and preferences are more likely to develop through interaction rather than abstract expectation alone. Research designs that capture evaluations following actual exposure to AI-generated scoring and feedback are, therefore, essential, yet such studies remain relatively scarce.

The present study addresses these gaps by examining undergraduate students’ perceptions of and preferences for AI-based scoring and feedback in higher education assessment, including evaluations formed after interaction with AI-generated outputs. By treating perceptions and preferences as distinct but related constructs and by grounding evaluations in lived experience rather than hypothetical scenarios, the study aims to provide a better understanding of how students interpret the role of AI in educational assessment. More specifically, it places student voice at the center of AI integration, recognizing that feedback can support learning only when students meaningfully engage with and use the information provided (Lipnevich et al., 2016). Specifically, the study addressed the following research questions (RQs):

1. What are students’ preferred characteristics of AI feedback, and do these preferences differ from chance?
2. How do students’ preferences for AI versus human scoring and feedback vary across (a) assessment scenarios and (b) types, and are these differences statistically significant?
3. What are students’ perceptions of AI scoring and feedback?
4. What are students’ preferences regarding (a) the role of AI and (b) AI-based scoring relative to human graders, and what factors predict these preferences?
5. What preferences do students express regarding the AI scoring and feedback after evaluating AI-generated assessment outputs in terms of (a) emerging themes and (b) sentiment and topic patterns?

## 2. Literature Review

### 2.1. The Integration of AI in Higher Education Assessment

AI is rapidly transforming higher education, affecting how universities deliver instruction, conduct assessment, and support student learning. Once limited to research or administrative use, AI now underpins many aspects of daily academic practice, including assessment and feedback, which return near-instant comments at scale (Hahn et al., 2021; Hockly, 2019). Universities are teaching larger and more diverse student populations across multiple delivery and assessment formats. This diversification creates four practical pressures: scale (i.e., managing hundreds or thousands of submissions), speed (i.e., providing timely feedback), personalization (i.e., meeting individual learning needs), and consistency (i.e., maintaining fair and reliable standards across cohorts and graders). AI scoring and feedback respond directly to these demands. They can process large volumes of work within minutes, return feedback almost instantly, and tailor guidance to common error patterns or to rubric criteria, while ensuring consistent application of standards (Al Harrasi & El Din, 2024; Yan et al., 2020; Zawacki-Richter et al., 2019). In practice, these tools transform assessment from a single event into a continuous learning cycle wherein students draft, receive immediate and specific feedback, revise, and resubmit. Instructors, meanwhile, can use dashboards to identify class-wide difficulties (e.g., weak evidence, missing units in calculations) and intervene sooner (Yan et al., 2020). This accelerated practice-feedback loop was described as a central advantage of AI-supported assessment, noting that immediacy allows students to engage with feedback. At the same time, their cognitive focus on the task remains high, thereby increasing opportunities for learning and revision (Thomas et al., 2026).

Two additional factors underscore the timeliness of AI scoring and feedback. First, diversified course delivery modes (e.g., entirely online, hybrid, intensive block formats) compress feedback windows. Rapid, rubric-aligned comments help maintain learner progress during limited contact periods (Zawacki-Richter et al., 2019). Second, increasing attention to equity and transparency has heightened student expectations for clear, consistent reasoning behind scores. Properly validated AI models can minimize rater drift and provide standardized explanations linked to assessment criteria, supporting both fairness reviews and accreditation processes (Al Harrasi & El Din, 2024; Yan et al., 2020). When paired with human oversight, this balance of speed, scale, personalization, and consistency enables educators to reallocate time from repetitive first-pass marking to higher-value coaching and rich feedback dialogue (Al Harrasi & El Din, 2024).

### 2.2. Conceptualizing Student Preferences

Understanding how students evaluate AI-supported assessment requires distinguishing between two related but conceptually distinct constructs: perceptions and preferences. Although these terms are sometimes used interchangeably in discussions of educational technology, they capture different dimensions of learners’ responses to AI-based systems. Student perceptions refer to learners’ evaluative beliefs and judgments regarding the characteristics of AI technologies, such as their usefulness, credibility, effectiveness, or fairness in educational contexts (Thomas et al., 2026). These perceptions reflect how students cognitively interpret and assess the performance and implications of AI systems when they encounter them in learning or assessment environments. Student preferences, by contrast, refer to learners’ orientations toward the incorporation of AI technologies into assessment processes. Rather than focusing on evaluative judgments about AI systems themselves, preferences capture students’ choices among alternative assessment arrangements, such as AI-generated scoring, human evaluation, or hybrid models that combine both sources of judgment (Kotlyar & Krasman, 2025; Nazaretsky et al., 2025).

This conceptual distinction is consistent with theoretical perspectives from technology acceptance research. The Technology Acceptance Model distinguishes between users’ beliefs about a system, such as perceived usefulness and ease of use, and their behavioural intentions regarding technology adoption (Davis, 1989). Similarly, the Unified Theory of Acceptance and Use of Technology suggests that individuals’ cognitive evaluations of technological systems influence, but do not fully determine, their willingness to adopt or rely on those technologies (Venkatesh et al., 2003). In the context of AI-based assessment, perceptions represent evaluative beliefs about AI systems, whereas preferences capture learners’ orientations toward how such systems should be integrated into assessment processes. Examining both perceptions and preferences, therefore, provides a more comprehensive understanding of how students interpret the role of AI in assessment and how they envision its integration into educational practice.

### 2.3. Perceptions and Preferences for AI-Based Scoring and Feedback

Research on student perceptions of AI in assessment has developed along two main lines. The first has examined technical performance: well-trained automated scoring models achieve consistency comparable to human raters, particularly in large-scale writing assessment, and machine-learning systems can be integrated into digital environments for scalable, rapid evaluation (Buzick et al., 2016; Flodén, 2025; Kumar & Boulanger, 2021). More recent work has extended this line of inquiry by deploying generative AI tools in live classroom settings. Stowell and Zhu (2025) developed a ChatGPT-powered application using retrieval-augmented generation to generate and grade essay questions in an undergraduate psychology course across four assessments. Agreement between AI and instructor scores was moderate overall (ICCs ranging from 0.21 to 0.81), with AI exhibiting a systematic tendency to avoid assigning extreme scores—grading lower-performing responses more generously and higher-performing ones more strictly than the human instructor.

A second line of research has examined students’ general attitudes toward generative AI tools in educational contexts. Survey-based studies suggest that students often recognize the potential benefits of AI technologies for learning. For example, Chan and Hu (2023) reported that university students generally viewed generative AI tools as useful for assisting with learning tasks such as generating explanations or supporting writing activities. Similarly, Ngo (2023) found that students perceived tools such as ChatGPT as helpful resources for academic work, particularly for idea generation and clarification of complex topics. At the same time, large-scale studies reveal a more nuanced picture. Kelly et al. (2023), based on a survey of more than 1000 university students, found that although many students expressed interest in using generative AI tools, they also reported limited knowledge and confidence in using them effectively. Likewise, Von Garrel and Mayer (2023) documented high levels of awareness of generative AI technologies among students but substantial variation in how frequently and for what purposes these tools were used.

Beyond general attitudes toward AI technologies, a growing number of studies have examined students’ evaluations of AI-generated feedback. These studies emphasize that the educational value of feedback depends not only on the information provided but also on how learners interpret and engage with that feedback. Educational research has long demonstrated that feedback supports learning only when students actively process and apply the information provided (Lipnevich et al., 2016). From this perspective, AI-supported feedback systems may influence students’ engagement with feedback processes by enabling more immediate and iterative forms of feedback interaction. Research on feedback literacy further highlights that effective feedback involves students actively seeking, understanding, and using feedback to improve their work (Carless & Boud, 2018; Molloy et al., 2020). Recent studies suggest that AI-enabled learning analytics and generative AI tools may support such processes by allowing students to request explanations, explore alternative responses, and iteratively refine their work (Jin et al., 2025).

While these lines of research provide valuable insights into students’ attitudes toward AI technologies and the technical capabilities of automated assessment systems, they offer only limited insight into how students envision the role of AI in assessment processes. In particular, existing research rarely examines how learners prefer specific characteristics of AI-generated feedback, such as tone, length, format, timing, or focus, to be structured, despite the importance of these features for the pedagogical effectiveness of feedback (Le et al., 2025; Zhan et al., 2025). Research shows that students can be more comfortable with AI scoring and feedback for low-stakes tasks and early drafts, notably when instructors frame AI as a supportive tool rather than a final authority (Al Harrasi & El Din, 2024; Nazaretsky et al., 2025). In contrast, for high-stakes or creative work, they favour human grading, citing trust in humans to interpret tone, originality, and meaning.

### 2.4. The Impact of Interaction with AI on Student Perceptions and Preferences

In existing studies, students are asked to respond to hypothetical scenarios involving AI-generated grading or feedback (Nazaretsky et al., 2025; Siregar et al., 2024). Such designs provide useful insights into students’ expectations about AI technologies but offer limited opportunities to examine how learners evaluate AI-generated feedback after directly interacting with these systems. As AI technologies become more embedded in educational environments, students are increasingly likely to encounter AI-supported assessment tools as part of their learning experiences.

Recent research suggests that direct interaction with AI systems can shape how learners perceive and evaluate AI technologies in educational contexts (Kasneci et al., 2023). When students engage with AI tools in learning activities, they can evaluate AI-generated outputs based on their perceived usefulness, clarity, and relevance rather than relying solely on prior expectations about AI technologies (Long & Magerko, 2020). Empirical studies illustrate how students’ perceptions evolve through such interactions. For example, Shoufan (2023) examined students’ perceptions of ChatGPT following their use of the system as part of a learning activity. The findings indicated that students generally viewed ChatGPT as a helpful learning tool that can support academic tasks, while also recognizing limitations in its accuracy and the need for sufficient background knowledge to evaluate AI-generated information critically. Stowell and Zhu (2025) offer particularly direct evidence of this pattern. In their study, a ChatGPT-powered web application generated and graded essay questions in an undergraduate psychology course, providing students with real-time, personalized feedback across four consecutive quizzes. Although students valued the immediacy and personalization of AI feedback, with 73% to 77% finding it helpful for learning, only 35% perceived the AI grading as fair, and 92% expressed a preference for instructor grading over AI-based scoring. These findings illustrate how direct exposure to AI-generated assessment can simultaneously reveal the practical value of AI feedback and deepen concerns about its fairness and legitimacy as a grading authority, a pattern that expectation-based survey designs are unlikely to capture.

Similar patterns were reported by Tossell et al. (2024), who investigated students’ perceptions of ChatGPT in the context of a university essay assignment requiring students to use the system during the writing process. Using a pre–post research design, the study found that students acknowledged the potential benefits of AI-supported tools for writing and learning tasks, yet expressed lower levels of trust in AI-generated grading compared with grading performed by human instructors. At the same time, many participants indicated support for hybrid evaluation approaches in which AI tools assist instructors rather than replace human judgment.

Generative AI tools enable learners to request explanations, ask follow-up questions, and iteratively revise their work based on generated responses. Such affordances may support more active forms of feedback engagement, including seeking, interpreting, and applying feedback to improve performance (Zhan et al., 2025). At the same time, research suggests that attribution effects may influence students’ evaluations of AI-generated outputs. When learners are informed that feedback was generated by AI rather than by a human instructor, they may evaluate the feedback more critically even when the content itself is identical (Nazaretsky et al., 2025). Empirical evidence further suggests that students often favour hybrid approaches that combine the efficiency and immediacy of AI systems with the contextual judgment and pedagogical expertise of human instructors (Le et al., 2025; Thomas et al., 2026). However, relatively few studies investigate how students believe AI systems should collaborate with human instructors within assessment practices, for example, as preliminary scorers, feedback assistants, or complementary evaluators.

## 3. Methods

### 3.1. Participants

This study employed a convenience sampling method, recruiting undergraduate students from the university’s research participation pool, who participated voluntarily in exchange for one percent extra course credit. Ethical approval for this study was obtained from the University of Alberta Research Ethics Board. Data collection was conducted at Concordia University of Edmonton. Informed consent and survey administration were conducted online through Qualtrics in Fall 2025, with participants completing the survey individually in a self-administered format. Participation was voluntary, with the option to withdraw at any time without penalty.

The sample consisted of 93 undergraduate students. Seventy-eight percent identified as female (*n* = 73), 19.3% as male (*n* = 18), and 2.2% as non-binary (*n* = 2). Participant age ranged from 17 to 40 (*M* = 22.06, *SD* = 5.33). Academic levels were distributed as follows: 33.3% were first-year students (*n* = 31), 17.2% were second-year students (*n* = 16), 30.1% were third-year students (*n* = 28), and 19.4% were fourth-year or higher (*n* = 18). Participants represented a variety of academic majors, with the largest group identifying as psychology majors (*n* = 70). The other majors included biology (*n* = 11), education (*n* = 2), French (*n* = 1), history (*n* = 1), management (*n* = 1), open studies (*n* = 2), science (*n* = 2), and sociology (*n* = 2). Table 1 summarizes background data about student knowledge of, experience with, and use of AI in an academic context.

### 3.2. Measure

Items for the survey were initially drafted based on constructs and subconstructs identified through a review of the existing literature (refer to the Appendix A, Appendix B, Appendix C, Appendix D, Appendix E, Appendix F and Appendix G). In addition to demographic and background questions as well as Likert-type and scenario-based items, participants completed a structured activity designed to elicit reflections on AI scoring and feedback. They were instructed to copy a short-answer question, a scoring rubric, and a brief student response into ChatGPT (OpenAI; GPT-4o/GPT-5-class; model version may vary due to system updates) and prompt the system to provide a score and feedback for this response based on the rubric. Then, participants were asked to reflect on the score and feedback.

Content validity of the survey was established through a review by one author, an expert in educational psychology with a research focus on AI. Cognitive validation was then conducted using a think-aloud protocol with a pilot participant. In this process, the participant read each question aloud while verbalizing their understanding and thought process for answering. Feedback from this session was incorporated to refine item wording. Internal consistency was calculated for multi-item Likert-type scales intended to measure the same underlying construct to assess the coherence of responses across items within each scale. It was not computed for single-item measures or item sets representing distinct or context-specific judgments that did not form a unified latent construct. All retained subscales demonstrated acceptable internal consistency, with Cronbach’s alpha values of 0.73 for AI scoring perceptions, 0.78 for AI feedback perceptions, 0.74 for AI preferences, and 0.77 for AI versus human preferences.

### 3.3. Data Analyses

The data analyses were conducted using R version 4.0.0 (R Core Team, 2020), a statistical programming environment widely used for data manipulation, visualization, and modelling. For survey data processing, the packages dplyr (Wickham et al., 2023) for data manipulation, tidyr (Wickham & Henry, 2020) for reshaping data, and ggplot2 (Wickham, 2016) for visualizing results were used. Likert-scale responses were analyzed and visualized using the likert package (Bryer & Speerschneider, 2016). For text-based analyses, including sentiment analysis and topic modelling, the tm package (Feinerer & Hornik, 2025) was employed for text cleaning and corpus management, SnowballC (Bouchet-Valat, 2023) for stemming, and topicmodels (Grün & Hornik, 2011) for Latent Dirichlet Allocation (LDA).

Regarding RQ1, frequency counts were reported to provide students’ preferences for the characteristics of AI-generated feedback (i.e., tone, length, format, timing, focus). To examine whether students’ preferences for different characteristics of AI-generated feedback differed from an equal distribution, chi-square goodness-of-fit tests were conducted for each feedback dimension (tone, length, format, timing, and focus).

RQ2 was addressed using two complementary sets of analyses: preferences across specific assessment scenarios visualized using a heatmap and preferences across assignment types–general, low-stakes, high-stakes, creative, and appeal-eligible assignments reported using frequency counts. Furthermore, to assess students’ preferences for AI involvement across different assignment scenarios and types, chi-square tests of independence were conducted. These tests evaluated whether the observed frequencies for the four scoring and feedback options (score and feedback by a human, score and feedback by AI, score by a human with AI feedback, score by AI with human feedback) differed significantly from an equal distribution, providing insight into the contexts in which students were more likely to prefer AI, human, or hybrid feedback approaches.

RQ3 and RQ4 were addressed using visualizations of Likert-type responses to summarize participants’ perceptions of AI scoring and feedback and their preferences for the role of AI relative to human graders. Negatively worded items were reverse-coded so that higher scores consistently reflected more favourable perceptions or preferences toward AI. In terms of preferences (RQ4), two ordinal logistic regression analyses were conducted to examine predictors of students’ preferences for AI scoring and feedback, as well as their relative preference for AI versus human scoring and feedback. The dependent variables were derived by calculating composite median scores across the 10 survey items assessing AI feedback preferences and the 10 survey items assessing AI relative to human grading. These composite scores were then categorized into three ordered levels: Low (median ≤ 2), Medium (median > 2 and ≤ 3), and High (median > 3). Predictor variables included comfort with technology, familiarity with AI, familiarity with AI scoring, familiarity with AI feedback, frequency of human feedback, and frequency of AI use.

Regarding RQ5, a thematic analysis was conducted on participants’ reflective responses to examine their preferences and perceptions regarding the use of AI in assessment. The analysis followed a reflexive thematic analysis approach (Braun & Clarke, 2006, 2019), in which responses were read iteratively to identify recurring patterns in participants’ positioning of AI. Coding emphasized preference-oriented stances toward AI use. The analysis was conducted by one primary coder, with themes refined through iterative review to ensure consistency and interpretive coherence. To enhance trustworthiness, an audit trail of coding decisions was maintained throughout the analysis process. In addition to the qualitative analysis, topic modelling (statistical extraction of latent themes from text data) was conducted to identify the main themes in the responses, and a context-specific sentiment analysis (computational identification of emotional tone in text) was conducted to identify the overall sentiment (i.e., positive or negative) expressed toward each theme.

## 4. Results

### 4.1. RQ1. Preferences for AI Feedback Characteristics

Participants’ preferences for AI-generated feedback emphasized flexibility, structure, and iterative support (see Table 2). Regarding the feedback tone, the most commonly selected option was a task-dependent mix (*n* = 39), indicating that students favour adaptable feedback styles over a single consistent tone. Regarding the length of feedback, participants most frequently preferred a combination of concise and detailed feedback (*n* = 34). Regarding format, bulleted points were the most preferred option (*n* = 40), indicating a clear preference for structured, easy-to-process feedback. For timing, participants most often selected feedback at multiple stages (*n* = 32). This highlights the importance of receiving feedback throughout the writing process rather than at a single endpoint. Finally, for feedback focus, participants most frequently preferred feedback on grammar and mechanics (*n* = 35).

Chi-square goodness-of-fit tests were conducted to examine whether participants’ preferences for AI-generated feedback characteristics differed from an equal distribution across response options. The results were significant for feedback tone (χ^2^(3) = 15.86, *p* = 0.001), feedback length (χ^2^(3) = 11.65, *p* = 0.009), feedback format (χ^2^(3) = 30.40, *p* < 0.001), and feedback focus (χ^2^(3) = 18.27, *p* < 0.001). However, for feedback timing, the chi-square test was not significant, χ^2^(3) = 6.57, *p* = 0.087, indicating that preferences for when feedback is received did not differ significantly from an equal distribution across options.

### 4.2. RQ2. Preferences for AI Versus Human Scoring and Feedback

#### 4.2.1. Preferences Across Scenarios

**Figure 1:**
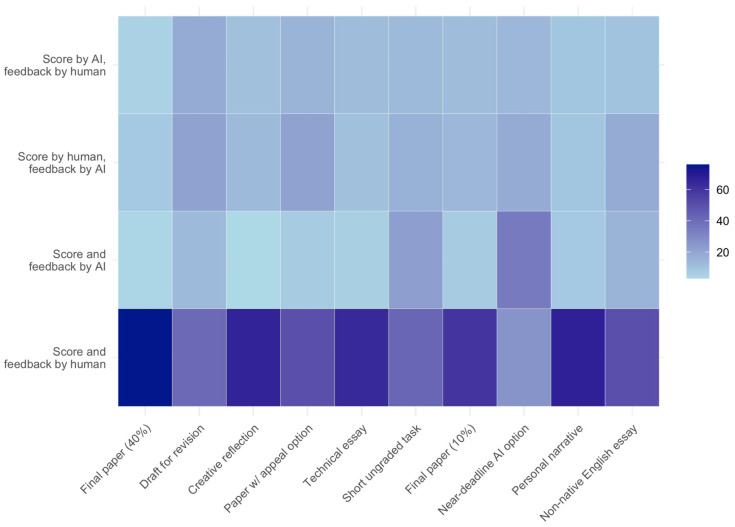
Heatmap showing the frequency of participant responses across scenarios. The full survey items are provided in the Appendix D.

Participant preferences for scoring and feedback across writing scenarios are shown in Table 3 and Figure 1. Participants indicated their preferred combination of scoring and feedback (human or AI) across ten assessment scenarios. Overall, the data show a strong preference for score and feedback provided entirely by a human, although preferences varied by task type and stakes. Across the scenarios, the majority of participants consistently chose human scoring and feedback for higher-stakes or summative tasks. For example, 76 participants preferred human scoring and feedback for the final paper (40% of course grade), 66 for the creative reflection, and 64 for the technical essay. Similarly, human scoring and feedback were favoured for the paper with appeal option (*n* = 50), final paper (10% of course grade; *n* = 60), and personal narrative (*n* = 67), reflecting a consistent trust in human judgment for assignments that contribute substantially to course grades or require expressive, interpretive responses.

AI-only scoring and feedback were the least preferred in most scenarios. Only four participants selected AI-only scoring for the final paper (40% of course grade), and the highest AI-only preference was observed for the near-deadline AI option (*n* = 35). Lower-stakes tasks, such as the short ungraded task, showed more openness to AI, with 22 participants selecting AI-only scoring and feedback and a combined 29 selecting hybrid approaches. These results suggest that students are more open to AI involvement in low-stakes or convenience-oriented tasks, especially when time pressure is a factor. Mixed approaches, in which scoring and feedback are split between humans and AI, showed moderate preference. Specifically, human scores and AI feedback received notable support for the draft for revision (*n* = 21), creative reflection (*n* = 13), and paper with appeal option (*n* = 21). Score by AI, feedback by a human was chosen by a smaller yet meaningful number of participants, particularly for drafts (*n* = 18), technical essay (*n* = 12), and final paper (10% of course grade) (*n* = 12).

A chi-square test of independence was conducted to examine whether preferences regarding scoring and feedback varied across assignment scenarios. The results indicated a significant association between scenario and preferred scoring/feedback method, χ^2^(27) = 134.57, *p* < 0.001. The effect size, as measured by Cramér’s V, was 0.22, indicating a small-to-moderate association between assignment context and scoring/feedback preferences. These findings suggest that participants’ choices of scoring and feedback methods were not uniform across scenarios but instead varied meaningfully by assignment characteristics (e.g., stakes, writing type, or context).

#### 4.2.2. Preferences Across Assignment Types

To further examine RQ2, participants also responded to a set of structured questions comparing preferences for AI versus human scoring and feedback across different assignment conditions, including general academic writing, low-stakes assignments, high-stakes assignments, creative or subjective writing, and assignments with the option to appeal or request a second review (see Table 4). Consistent with the scenario-based results, participants showed a strong overall preference for human involvement in scoring and feedback across academic writing assignments. The majority favoured both scoring and feedback by a human, with particularly high support for high-stakes assignments (*n* = 79) and general assignments (*n* = 74). Fully AI-based scoring and feedback were rarely preferred, with the number of respondents selecting this option ranging from 1 to 7 across the five questions. Mixed scoring and feedback options were more commonly selected for low-stakes assignments (*n* = 36).

A chi-square test of independence was conducted to examine whether preferred scoring and feedback methods differed across assignment types. The results indicated a significant association between assignment type and preferred scoring/feedback method, χ^2^(12) = 35.19, *p* < 0.001, with a small effect size, Cramér’s V = 0.16. Given that some expected cell counts were low, results should be interpreted with caution.

### 4.3. RQ3. Perceptions of AI Scoring and Feedback

**Figure 2:**
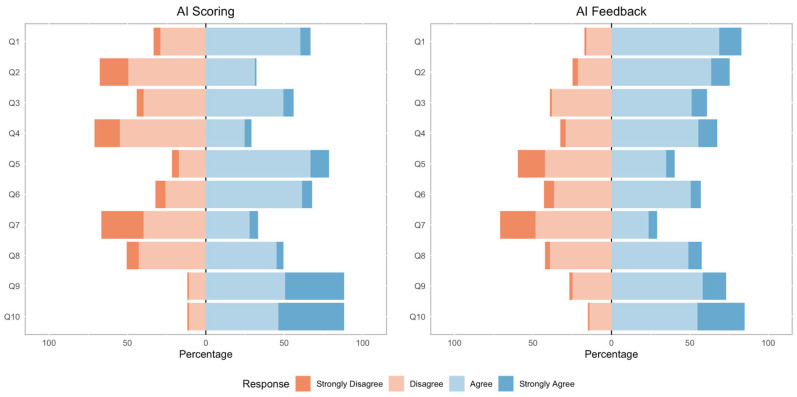
Perceptions of AI scoring (**left**) and AI feedback (**right**). The full survey items are provided in the Appendix E. (**Left panel**): Q1: objectivity; Q2: consistency; Q3: transparency; Q4: fairness across styles; Q5: grammar/mechanics accuracy; Q6: clarity/organization accuracy; Q7: creative writing accuracy; Q8: score consistency across similar work; Q9: overlooks key aspects; Q10: tone/emotion interpretation. (**Right panel**): Q1: clarity; Q2: next steps; Q3: strengths; Q4: improvements; Q5: avoids vagueness; Q6: content relevance; Q7: supports independence; Q8: generality; Q9: repetition; Q10: contradictions.

**Figure 3:**
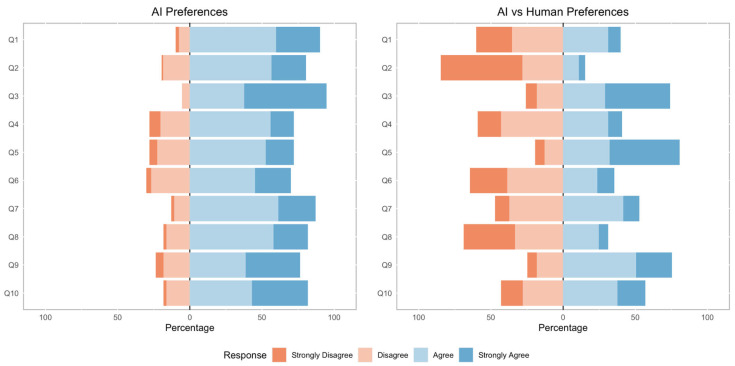
Preferences for AI (**left**) and AI relative to humans (**right**). The full survey items are provided in the Appendix F. (**Left panel**): Q1: AI grading breakdown; Q2: confidence levels; Q3: opt-in/opt-out of AI grading; Q4: rubric justification; Q5: transparency of AI decisions; Q6: feedback without scores; Q7: strengths/weaknesses feedback; Q8: specific examples; Q9: learning resources; Q10: follow-up questions. (**Right panel**): Q1: AI vs. human for low-stakes grading; Q2: AI vs. human for high-stakes grading; Q3: human review of AI scoring; Q4: AI feedback with human scores; Q5: AI assists vs. replaces humans; Q6: equal AI–human weighting; Q7: AI for pre-submission self-assessment; Q8: AI in final grading vs. human only; Q9: side-by-side AI vs. human comparison; Q10: AI feedback before human review.

While Figure 2 and Figure 3 present the full distribution of responses across the four Likert-type categories, the results are summarized in the text using aggregated agreement (Agree and Strongly Agree) and disagreement (Disagree and Strongly Disagree).

For AI scoring (refer to the left panel in Figure 2), most agreed that it is objective (Q1; agreement = 62 vs. disagreement = 31), transparent (Q3; 52 vs. 41), and accurate in grading grammar and mechanics (Q5; 73 vs. 20) as well as organization and clarity (Q6; 63 vs. 30). Fewer agreed that scoring is consistent (Q2; 30 vs. 63), fairly evaluates diverse writing styles (Q4; 27 vs. 66), or accurately assesses complex or creative writing (Q7; 31 vs. 62). Responses were evenly split on whether AI assigns similar scores to similar-quality work (Q8; 45 vs. 46). Many participants expressed concerns that AI grading might overlook important aspects (Q9; 82 vs. 11) or struggle to interpret tone and emotion (Q10; 82 vs. 11).

For AI feedback (refer to the right panel in Figure 2), most agreed that it is easy to understand (Q1; agreement = 77 vs. disagreement = 16), provides clear next steps (Q2; 70 vs. 23), and identifies strengths (Q3; 56 vs. 36) and areas for improvement (Q4; 62 vs. 30). Feedback was also seen as relevant to content (Q6; 53 vs. 40). Fewer participants agreed that it avoids vague comments (Q5; 37 vs. 55) or fosters independence as a writer (Q7; 27 vs. 66), while many noted that feedback can be too general (Q8; 53 vs. 39), repetitive (Q9; 68 vs. 25), or occasionally contradictory (Q10; 79 vs. 14).

### 4.4. RQ4. Preferences for AI and AI Relative to Human Graders

#### 4.4.1. Preferences for AI Scoring and Feedback

Participants expressed clear preferences regarding AI scoring and feedback (refer to the left panel in Figure 3). Most favoured greater control, including seeing a breakdown of how AI grades their writing (Q1; agreement = 83 vs. disagreement = 9), receiving confidence levels for AI scores (Q2; 74 vs. 18), opting in or out of AI grading (Q3; 88 vs. 5), and receiving rubric-based justifications (Q4; 67 vs. 26). Similar agreement was observed for preferences that AI decision-making be fully transparent (Q5; 67 vs. 26), that AI provide feedback without assigning scores (Q6; 65 vs. 28), and that feedback highlight both strengths and weaknesses (Q7; 81 vs. 12). Participants also largely preferred feedback referencing specific examples (Q8; 76 vs. 17), including links or resources for further learning (Q9; 71 vs. 22), and allowing follow-up questions about scores and feedback (Q10; 76 vs. 17).

An ordinal logistic regression (see Table 5) was conducted to examine predictors of students’ preferences for AI-based scoring and feedback. To address sparse response categories and potential separation in the original human feedback frequency variable, human feedback frequency categories were collapsed into three levels: frequent (daily and weekly), monthly, and infrequent (occasionally and rarely/never). The model demonstrated improved fit compared with the intercept-only model, with a residual deviance of 128.62 and an AIC of 168.62. Frequency of AI use was a significant predictor of AI preference. Specifically, students who rarely or never used AI had higher odds of being in a higher category of AI preference compared with the reference group, *B* = 2.11, *SE* = 1.03, *t* = 2.05, *p* = 0.040, OR = 8.24. No other predictors, including comfort with technology, familiarity with AI, familiarity with AI scoring, familiarity with AI feedback, and frequency of receiving human feedback, reached statistical significance at the α = 0.05 level.

#### 4.4.2. Preferences for AI Relative to Human Grading

Regarding preferences for AI relative to human grading (refer to the right panel in Figure 3), participants were less likely to prefer AI over a human grader for low-stakes assignments (Q1; agreement = 37 vs. agreement = 56) and strongly opposed AI for high-stakes grading (Q2; 14 vs. 78). Most preferred AI scoring and feedback to be reviewed by a human (Q3; 69 vs. 24) and favoured AI assisting rather than replacing human graders (Q5; 75 vs. 18). Fewer preferred AI-generated feedback with human-assigned scores (Q4; 38 vs. 55) or equal weighting of AI and human evaluation (Q6; 33 vs. 60). Preferences were more evenly split for using AI for fast self-assessment before submission (Q7; 48 vs. 43) and receiving AI feedback before human review to maximize speed and insight (Q10; 53 vs. 40). Most supported side-by-side comparisons of AI and human scoring (Q9; 70 vs. 23), while fewer favoured AI involvement in final grading over exclusive human evaluation (Q8; 29 vs. 64).

An ordinal logistic regression (see Table 6) was conducted to examine predictors of students’ preferences for AI-based scoring and feedback relative to human grading. The model demonstrated improved stability, with a residual deviance of 147.85 and an AIC of 187.85. Comfort with technology and frequency of receiving human feedback emerged as significant predictors. Students who were somewhat uncomfortable with technology had lower odds of being in a higher AI preference category compared with students in the reference category, B = −1.37, SE = 0.68, t = −2.01, *p* = 0.045, OR = 0.26. In addition, students who received human feedback frequently had higher odds of being in a higher category of preference for AI-based scoring and feedback compared with students receiving monthly feedback, B = 1.24, SE = 0.58, t = 2.13, *p* = 0.033, OR = 3.45. No other predictors, including familiarity with AI, familiarity with AI scoring, familiarity with AI feedback, and frequency of AI use, were statistically significant (all *p*s > 0.05).

### 4.5. RQ5. Post-Evaluation Preferences for AI Scoring and Feedback

#### 4.5.1. Results from Thematic Analysis

A total of 63 participants responded to the reflection question. Thematic analysis of reflections revealed four distinct preference-oriented themes regarding AI scoring and feedback: AI as a formative learning aid, preference for human authority, conditional or context-dependent acceptance, and skepticism about its educational impact (refer to Table 7). Each response was coded with a maximum of two themes to capture dominant preferences while avoiding over-coding. Most respondents expressed nuanced positions rather than absolute support or rejection.

Theme 1. The most prevalent theme was the use of AI as a learning support tool, particularly for feedback, revision, or improvement prior to final submission. Participants frequently emphasized the value of speed and guidance, describing AI as useful for “quick responses” and “instant feedback.” Several respondents highlighted its usefulness for improvement, noting that it can help students see “ways to improve” or “what needs improving.”

Theme 2. A substantial number of participants expressed a clear preference for human judgment over AI, particularly for final or subjective grading. These respondents emphasized the importance of nuance, emotion, and personal understanding, stating that “human scoring and feedback should never be replaced.” Others noted that human graders can “connect more” with student responses or apply “discretion” that AI lacks.

Theme 3. Some respondents supported AI use only under specific conditions, indicating that acceptance depended on the assignment’s type or stakes. These participants explicitly limited AI’s role, stating it was appropriate for “low-risk assignments,” “little assignments,” or tasks that are “not personal,” but not for “creative expression” or final evaluations.

Theme 4. A substantial number of participants expressed concerns about the broader implications of AI use in education, even when acknowledging its usefulness. Common issues included inconsistency, lack of depth, and negative effects on learning, with respondents describing feedback as “too vague “or “not clear,” and expressing worry that AI could reduce “critical thinking.” Others raised concerns about fairness and reliability, noting that AI “cannot be trusted 100%” or may grade differently across attempts.

#### 4.5.2. Results from Sentiment Analysis and Topic Modelling

A context-specific sentiment analysis was conducted to examine the overall tone of participants’ reflections regarding AI-generated scoring and feedback. Sentiment analysis is a computational technique used to identify positive and negative sentiment expressed in text. A custom lexicon was developed for the context of AI feedback and assessment, consisting of positive terms (e.g., clear, constructive, useful, helpful, accurate, detailed, reliable, and efficient) and negative terms (e.g., vague, confusing, inconsistent, impersonal, unreliable, incomplete, and repetitive). Because the sentiment lexicon was developed specifically for the context of AI-generated assessment feedback, the identified terms were selected based on their relevance to the study context rather than drawn from a standardized sentiment database. For each response, the frequency of positive and negative words was calculated, and an overall sentiment score was computed as the difference between the two counts (see Table 8). Positive scores indicated more favourable sentiment, whereas negative scores indicated more critical sentiment toward AI-generated scoring and feedback.

Regarding the topic modelling, LDA was used to identify themes within the student responses. A document-term matrix was constructed, and an LDA model with three topics was fitted. The top terms for each topic were manually inspected, and descriptive labels were assigned based on recurring themes. The interpretation and labeling of topics involved researcher judgment; therefore, topic assignments should be considered an analytical interpretation of response patterns. The topics were (1) detailed feedback and examples, (2) usefulness and clarity of feedback, and (3) accuracy of scoring and feedback. Each response was assigned to the topic with the highest probability from the LDA model. Aggregated sentiment scores for each topic are presented in Table 8. Across all topics, positive sentiment-related terms occurred more frequently than negative terms, resulting in higher positive sentiment scores.

## 5. Discussion

This study examined undergraduate students’ perceptions of and preferences for AI-based scoring and feedback in educational assessment, with particular attention to when students prefer AI involvement, how they believe AI should function relative to human graders, and how evaluating AI-generated scoring and feedback relates to these preferences. Overall, the findings suggest that students recognize the practical value of AI for efficiency, revision support, and formative learning, but continue to strongly prefer human evaluators for final judgment, especially in high-stakes, subjective, and complex assessment contexts. These results reinforce the growing literature suggesting that acceptance of AI in education depends not only on technical performance but also on trust, transparency, and the perceived appropriateness of AI within specific pedagogical contexts (Dietvorst et al., 2015; Gaube et al., 2021; Nazaretsky et al., 2025).

### 5.1. RQ1. Preferences for AI Feedback Characteristics

Regarding students’ preferences for the characteristics of AI-generated feedback, participants clearly favoured structured, adaptable, and useful feedback for revision. Students most often preferred a mixed tone depending on the task, a combination of concise and detailed feedback, bulleted formatting, and feedback focused primarily on grammar and mechanics. These findings align with prior research showing that students value feedback that is immediately actionable, easy to process, and directly connected to improvement (Le et al., 2025; Zhan et al., 2025). The preference for bulleted feedback and combined concise-detailed responses suggests that students seek both efficiency and depth in feedback that is quick to interpret but still sufficiently explanatory to support revision. Similarly, the preference for grammar and mechanics reflects students’ recognition that AI performs particularly well in lower-order writing concerns, consistent with studies demonstrating stronger AI performance in technical and linguistic correction than in higher-order rhetorical or creative evaluation (Buzick et al., 2016; Flodén, 2025). Interestingly, feedback timing did not differ significantly from chance; however, students valued feedback opportunities across multiple stages rather than privileging one specific moment. This supports research on feedback literacy emphasizing iterative feedback cycles rather than single-point evaluative events (Carless & Boud, 2018; Molloy et al., 2020).

### 5.2. RQ2. Preferences for AI Versus Human Scoring and Feedback

Students’ preferences for AI versus human scoring and feedback varied meaningfully across assignment contexts. AI-only scoring/feedback or mixed scoring/feedback options (i.e., score by AI with human feedback or vice versa) were chosen less frequently, though slightly more in low-stakes or time-constrained situations. Participants strongly favoured having both the score and feedback provided by a human, particularly for high-stakes or complex tasks, such as final papers (40% or 10% of the course grade), creative reflection, technical essays, and personal narratives. In less critical or time-sensitive scenarios, such as short ungraded tasks or near-deadline drafts, participants still preferred human feedback. However, a small portion of responses indicated openness to AI scoring and feedback or hybrid approaches. These findings are consistent with previous research suggesting that students are more comfortable with AI involvement when the perceived academic risk is low and when AI is framed as a supportive rather than authoritative tool (Al Harrasi & El Din, 2024; Nazaretsky et al., 2025). The strong preference for human evaluators in high-stakes and creative assignments likely reflects trust in human capacity for nuance, interpretation, and contextual judgment—qualities students may perceive AI as lacking. This also aligns with broader findings from algorithm aversion research, in which individuals prefer human judgment in subjective domains even when algorithmic systems demonstrate strong objective performance (Dietvorst et al., 2015).

### 5.3. RQ3. Perceptions of AI Scoring and Feedback

Participants’ perceptions of AI scoring and feedback further help explain these preferences. Students generally viewed AI positively for objective and technical assessment functions. Most agreed that AI scoring was accurate for grammar, mechanics, and organization, and many viewed it as objective and relatively transparent. However, participants were considerably more skeptical about AI’s ability to evaluate diverse writing styles, creative work, and complex or subjective responses. Many also believed AI might overlook important aspects of writing or struggle to interpret tone and emotion. Similarly, while AI feedback was viewed as understandable, clear, and helpful for identifying strengths and weaknesses, students also described it as repetitive, vague, and sometimes contradictory. These findings reflect the distinction between lower-order and higher-order feedback functions reported in prior literature (Fu et al., 2022; Wilson et al., 2021). Students appear willing to trust AI when evaluation involves rule-based or standardized criteria, but remain cautious when assessment requires interpretation, originality, or disciplinary nuance. This supports the argument that positive perceptions of AI usefulness do not necessarily translate into a preference for AI authority in assessment decisions (Gaube et al., 2021; Kasneci et al., 2023).

### 5.4. RQ4. Preferences for AI and AI Relative to Human Graders

The findings are consistent with prior work demonstrating that students perceive AI systems as fairer when explanations are provided and when users retain agency in how AI is used (Chai et al., 2024; Nazaretsky et al., 2025). Students expressed strong preferences for transparency, control, and human oversight when AI is involved in assessment. Most participants wanted the option to opt in or out of AI grading, see how AI-generated scores, receive confidence levels for AI judgments, and access rubric-based explanations. They also preferred AI feedback that referenced specific examples and allowed follow-up questions. These findings highlight that students do not simply evaluate AI based on outcomes, but also on process visibility and perceived accountability. Transparency appears central to trust formation, particularly in contexts where AI influences academic outcomes. Importantly, participants preferred AI-assisted rather than AI-replaced human graders, and strongly supported human review of AI-generated scoring and feedback. This indicates that hybrid models may represent the most pedagogically acceptable approach, balancing efficiency with human judgment and relational trust.

Experience with human feedback may shape students’ perceptions of assessment quality by influencing their expectations regarding personalized, contextualized, and relational aspects of feedback. Frequency of receiving human feedback emerged as a significant predictor, with students who received frequent human feedback showing higher odds of preferring AI-based scoring and feedback compared with students receiving monthly feedback. This finding suggests that students who frequently engage with feedback processes may be more open to considering AI-based assessment tools as complementary forms of support. Comfort with technology also predicted relative preference for AI, with students who were somewhat uncomfortable with technology showing significantly lower preference for AI over human grading. This finding aligns with acceptance models emphasizing digital self-efficacy as a key predictor of technology adoption (Davis, 1989; Venkatesh et al., 2003). Interestingly, general familiarity with AI and frequency of AI use were not significant predictors, suggesting that familiarity alone may not drive acceptance of AI in assessment. Rather, students’ comparative experiences with human feedback and their confidence in navigating technological systems may be more influential.

### 5.5. RQ5. Post-Evaluation Preferences for AI Scoring and Feedback

Students’ reflections following their evaluation of AI-generated scoring and feedback further supported these patterns. The most common theme positioned AI as a formative learning aid rather than a final evaluator. Participants valued AI for quick feedback, revision support, and identifying areas for improvement, reinforcing the idea that students see AI as most useful during drafting and revision stages. At the same time, many emphasized the importance of human authority for final judgment, particularly in subjective or high-stakes assessments. Others expressed conditional acceptance with findings that closely mirror the quantitative results, suggesting that interaction with AI reinforces nuanced rather than polarized views. Students were not wholly supportive or opposed to AI; instead, they advocated for bounded, context-sensitive integration. This is consistent with prior pre–post research showing that students support hybrid systems more strongly after direct engagement with AI tools (Tossell et al., 2024).

Interestingly, sentiment analysis showed that overall reflections were more positive than negative, particularly regarding usefulness, clarity, and detailed feedback. Topic modelling further emphasized students’ attention to the quality of practical feedback rather than abstract concerns about AI itself. This suggests that students evaluate AI largely through its pedagogical usefulness rather than through purely ideological acceptance or rejection. Even students who expressed concerns about AI often acknowledged its value as a learning support tool. This reinforces the importance of distinguishing between perceptions and preferences. Students may perceive AI positively yet prefer human evaluators for final assessment decisions.

Students’ preferences and perceptions of AI may evolve with experience, but the findings suggest that this change is not driven simply by increased familiarity with AI tools. In this study, familiarity with AI use was not a significant predictor of preferences, indicating that repeated exposure alone may not substantially shift attitudes toward AI in assessment. However, results from the post-interaction phase show that direct engagement with AI feedback led to more nuanced and, in some cases, more positive evaluations, particularly regarding its usefulness for revision. This suggests that meaningful, task-specific interaction with AI may be more influential than general familiarity.

## 6. Conclusions

This study contributes to the growing literature on AI in higher education by demonstrating that students’ preferences for AI-based scoring and feedback are highly context-dependent. Students value AI for speed, clarity, and formative support, particularly in grammar-focused and low-stakes assessment contexts, but strongly prefer human evaluators for high-stakes, subjective, and complex assignments. Transparency, control, and human oversight emerged as central conditions for trust, while the relationship between experience with human feedback and AI preference was more nuanced, with frequent human feedback associated with higher AI preference in one model and not emerging as a significant predictor in another. Rather than seeking full automation, students favoured hybrid models in which AI assists rather than replaces human judgment. These findings suggest that the future of AI in educational assessment lies not in substitution, but in carefully designed collaboration between human expertise and AI.

### 6.1. Implications

These findings have important implications for the integration of AI in higher education assessment. First, AI feedback should focus on being clear, structured, and easy to use for revision, rather than trying to be overly detailed or complex. Second, AI is better suited as a support tool in low-stakes or early stages of assignments, while human teachers should remain responsible for final evaluation, especially in high-stakes or complex tasks. Third, AI should be mainly used for technical aspects such as grammar and organization, since students are less confident in its ability to judge creativity or interpretation. Furthermore, it is important that AI systems are open and understandable, with human oversight kept in place to ensure trust and accountability. Student responses also suggest that AI is most useful as a learning and revision tool, so it should be integrated into drafting and improvement stages rather than final grading decisions.

The findings also suggest that institutions should avoid positioning AI as a replacement for human grading and instead adopt hybrid models in which AI supports formative feedback, early drafting, and lower-stakes evaluation, while human instructors retain responsibility for final judgment, especially in high-stakes and subjective tasks. This approach aligns more closely with student preferences and may reduce resistance to AI adoption. The strong influence of the frequency of human feedback suggests that AI implementation should complement, not displace, meaningful instructor feedback. Preserving opportunities for relational and personalized feedback remains critical for student trust and educational effectiveness.

Transparency should also be treated as a core principle. Students want to understand how AI reaches evaluative decisions and expect opportunities to question or challenge those outcomes. Systems that provide rubric-linked justifications, confidence indicators, and opportunities for follow-up may improve trust and acceptance. Furthermore, since students value feedback across multiple stages rather than a single point, instructors could integrate AI in iterative submission cycles (draft → AI feedback → revision → human evaluation) rather than one-off grading use.

Digital literacy and AI literacy initiatives remain essential. Students who are less comfortable with technology were less likely to prefer AI involvement, suggesting that institutions should support students not only in using AI tools but also in critically understanding their strengths and limitations. Such literacy may reduce both overreliance and unnecessary skepticism.

### 6.2. Limitations and Future Directions

Several limitations should be considered. The study relied on a convenience sample from a single university, with a large proportion of psychology students and female participants, which may limit generalizability across disciplines and institutional contexts. Therefore, the findings should be interpreted as reflecting the perceptions of this specific student population and may not generalize to students from other disciplines, institutions, or demographic backgrounds. Future research should extend this study to additional institutions to improve the generalisability of the findings. In addition, future research should examine whether preferences differ across fields in which assessment norms vary substantially, such as engineering, the fine arts, or professional programs. It also focused primarily on self-reported preferences and perceptions rather than long-term behavioural outcomes. Future research could examine how students actually use AI-generated feedback across a semester and whether preferences change with sustained exposure rather than a single interaction.

The findings should also be interpreted in light of the sample size and model complexity. Although the ordinal regression models provided insights into factors associated with students’ AI assessment preferences, the relatively small sample size and the number of estimated parameters may have limited the stability and generalizability of the regression estimates. Therefore, the reported associations should be considered exploratory and warrant further investigation with larger samples and more parsimonious models. Furthermore, given the number of statistical comparisons conducted across feedback dimensions and assessment scenarios, the possibility of inflated Type I error should be considered. Because these analyses were exploratory in nature, results from individual chi-square tests, particularly those with marginal significance levels or smaller expected cell counts, should be interpreted cautiously. Future studies with larger samples and preregistered hypotheses may help confirm the observed patterns.

Although ChatGPT was used as the AI tool for interaction, AI systems vary considerably in sophistication and design. Future studies should compare multiple AI platforms and feedback interfaces to determine whether preferences are shaped by the technology itself or by broader beliefs about AI. Future studies should also compare ChatGPT with other AI tools such as Gemini, Claude, DeepSeek, and Copilot to enable cross-system evaluation of AI-supported assessment. A further limitation is that participants evaluated AI-generated responses to a common prompt rather than receiving feedback on their own writing, which may reduce the ecological validity of the task. Future studies should examine student perceptions using feedback generated on participants’ own coursework or authentic assessment tasks. Additionally, because AI-generated responses can vary across interactions, some variability in participants’ experiences may have occurred despite the use of a standardized prompt. Future research should explore the extent to which variability in AI-generated responses influences student perceptions and evaluation of AI-supported assessment. Furthermore, faculty perspectives were not included. Given that instructors play a central role in framing and implementing AI-supported assessment, future work should examine faculty beliefs about AI in assessment and how these interact with student preferences to shape acceptance of AI in educational environments.

Although qualitative responses were systematically analyzed using thematic coding, topic modeling, and sentiment analysis procedures, these analyses involved researcher judgment in coding, interpreting topic structures, and developing the sentiment lexicon. Coding and interpretation were conducted by a single researcher, and inter-coder reliability was not assessed. Additionally, the sentiment analysis was based on a researcher-developed lexicon and frequency patterns of sentiment-related terms, which may not fully capture the contextual meaning of students’ responses. Therefore, these findings should be interpreted as exploratory patterns, and future studies should incorporate multiple independent coders and validated sentiment analysis approaches to further establish the reliability and validity of qualitative findings.

## Figures and Tables

**Table 1:** Distribution of responses for technology comfort, AI familiarity, and use.

Variable	Response Category	% (*n*)
Comfort with technology	Very uncomfortable	10.8 (10)
	Somewhat uncomfortable	14.0 (13)
	Somewhat comfortable	51.6 (48)
	Very comfortable	23.7 (22)
Familiarity with AI	Not familiar at all	3.2 (3)
	Not very familiar	14.0 (13)
	Somewhat familiar	62.4 (58)
	Very familiar	20.4 (19)
Familiarity with AI scoring	Not familiar at all	19.4 (18)
	Not very familiar	41.9 (39)
	Somewhat familiar	33.3 (31)
	Very familiar	5.4 (5)
Familiarity with AI feedback	Not familiar at all	15.1 (14)
	Not very familiar	38.7 (36)
	Somewhat familiar	37.6(35)
	Very familiar	8.6 (8)
Frequency of receiving human feedback	Daily	2.2 (2)
	Weekly	35.5 (33)
	Monthly	34.4 (32)
	Occasionally	24.7 (23)
	Rarely/Never	3.2 (3)
Frequency of AI use	Daily	9.7 (9)
	Weekly	28.0 (26)
	Monthly	10.8 (10)
	Occasionally	31.2 (29)
	Rarely/Never	20.4 (19)
Purpose of AI use	Enhancing learning and understanding	66.7 (62)
	Summarizing course content or materials	53.8 (50)
	Proofreading, correcting grammar, spelling, & punctuation	47.3 (44)
	Generating ideas or brainstorming	49.5 (46)
	Writing drafts or essays	9.7 (9)
	Language translation	28 (26)

Note: For “Purpose of AI use,” participants could select multiple responses; therefore, percentages may exceed 100%.

**Table 2:** Preferences for characteristics of AI-generated feedback.

Feedback Dimension	Response Option	% (*n*)
Tone	Encouraging	14.0 (13)
	Neutral	22.6 (21)
	Critical	21.5 (20)
	A mix depending on the task	41.9 (39)
Length	Concise (short, to-the-point suggestions)	14.0 (13)
	Detailed (thorough explanations and examples)	30.1 (28)
	A combination of both (key points & detailed guidance)	36.6 (34)
	Depends on the assignment (concise for low-stakes, detailed for high-stakes)	19.4 (18)
Format	Bulleted points	43.0 (40)
	Paragraph-style	7.5 (7)
	Inline comments on the text	15.1 (14)
	A mix of formats	34.4 (32)
Timing	After the first draft	26.9 (25)
	After revisions	16.1 (15)
	Before submission	22.6 (21)
	At multiple stages	34.4 (32)
Focus	Content and ideas	24.7 (23)
	Organization and structure	30.1 (28)
	Grammar and mechanics	37.6 (35)
	Style and tone	7.5 (7)

**Table 3:** Participants’ preferred scoring and feedback across assessment scenarios.

Scenario	Human Score & Feedback	AI Score & Feedback	Human Score & AI Feedback	AI Score & Human Feedback
Final paper (40%)	76 (81.7%)	4 (4.3%)	8 (8.6%)	5 (5.4%)
Draft for revision	41 (44.1%)	13 (14.0%)	21 (22.6%)	18 (19.4%)
Creative reflection	66 (71.0%)	3 (3.2%)	13 (14.0%)	11 (11.8%)
Paper with an appeal option	50 (53.8%)	7 (7.5%)	21 (22.6%)	15 (16.1%)
Technical essay	64 (68.8%)	6 (6.5%)	11 (11.8%)	12 (12.9%)
Short ungraded task	42 (42.5%)	22 (23.7%)	16 (17.2%)	13 (14.0%)
Final paper (10%)	60 (64.5%)	7 (7.5%)	14 (15.1%)	12 (12.9%)
Near-deadline AI option	26 (28.0%)	35 (37.6%)	18 (19.4%)	14 (15.1%)
Personal narrative	67 (72.0%)	8 (8.6%)	9 (9.7%)	9 (9.7%)
Non-native English essay	50 (53.8%)	15 (16.1%)	18 (19.4%)	10 (10.8%)

**Table 4:** Preferred scoring and feedback across assignment types.

Assignment Type	Human Score & Feedback	AI Score & Feedback	Human Score & AI Feedback	AI Score & Human Feedback
General academic writing	74 (79.6%)	2 (2.2%)	9 (9.7%)	8 (8.6%)
Low-stakes assignments	50 (53.8%)	7 (7.5%)	17 (18.3%)	19 (20.4%)
High-stakes assignments	79 (84.9%)	1 (1.1%)	7 (7.5%)	6 (6.5%)
Creative/subjective writing	66 (71.0%)	4 (4.3%)	17 (18.3%)	6 (6.5%)
Assignments with an appeal option	63 (67.7%)	4 (4.3%)	19 (20.4%)	7 (7.5%)

**Table 5:** Ordinal logistic regression predicting AI preference.

Predictor	*B*	*SE*	*t*	*p*	OR
Comfort with technology					
Very uncomfortable	1.28	1.17	1.09	0.276	3.59
Somewhat uncomfortable	−0.59	0.74	−0.81	0.419	0.55
Very comfortable	−1.20	0.63	−1.90	0.058	0.30
Familiarity with AI (general)					
Not very familiar	0.60	1.52	0.39	0.695	1.82
Somewhat familiar	−0.17	1.50	−0.11	0.911	0.85
Very familiar	0.58	1.63	0.36	0.720	1.79
Familiarity with AI scoring					
Not very familiar	0.41	1.14	0.36	0.717	1.51
Somewhat familiar	−0.15	1.23	−0.12	0.902	0.86
Very familiar	0.44	1.81	0.24	0.807	1.56
Familiarity with AI feedback					
Not very familiar	0.31	1.12	0.27	0.785	1.36
Somewhat familiar	1.23	1.22	1.00	0.316	3.41
Very familiar	0.05	1.60	0.03	0.974	1.05
Frequency of receiving human feedback					
Frequent	0.35	0.62	0.55	0.580	1.41
Infrequent	0.52	0.69	0.75	0.455	1.68
Frequency of AI use					
Weekly	1.28	0.96	1.33	0.184	3.59
Monthly	1.87	1.15	1.63	0.103	6.50
Occasionally	1.24	0.92	1.35	0.179	3.46
Rarely/Never	2.11	1.03	2.05	0.040 *	8.24

* for *p* < 0.05. *B* = ordinal logistic regression coefficient; *SE* = standard error; *OR* = odds ratio. Positive coefficients indicate higher odds of being classified into a higher category of preference for AI-based scoring and feedback.

**Table 6:** Ordinal logistic regression predicting AI preference relative to human scoring and feedback.

Predictor	*B*	*SE*	*t*	*p*	OR
Comfort with technology					
Very uncomfortable	−0.77	0.81	−0.95	0.343	0.47
Somewhat uncomfortable	−1.37	0.68	−2.01	0.045 *	0.26
Very comfortable	−0.85	0.61	−1.40	0.161	0.43
Familiarity with AI (general)					
Not very familiar	−0.01	1.36	−0.01	0.994	0.99
Somewhat familiar	0.54	1.35	0.40	0.688	1.72
Very familiar	−0.67	1.45	−0.46	0.644	0.51
Familiarity with AI scoring					
Not very familiar	−0.58	1.02	−0.57	0.569	0.56
Somewhat familiar	−0.05	1.09	−0.04	0.964	0.95
Very familiar	1.60	1.81	0.88	0.376	4.96
Familiarity with AI feedback					
Not very familiar	−0.21	0.99	−0.21	0.836	0.81
Somewhat familiar	0.50	1.09	0.46	0.646	1.65
Very familiar	−0.29	1.51	−0.19	0.847	0.75
Frequency of receiving human feedback					
Frequent	1.24	0.58	2.13	0.033 *	3.45
Infrequent	−0.38	0.62	−0.61	0.545	0.69
Frequency of AI use					
Weekly	−0.03	0.91	−0.04	0.970	0.97
Monthly	0.53	1.09	0.49	0.625	1.70
Occasionally	−0.08	0.86	−0.09	0.930	0.93
Rarely/Never	0.34	0.95	0.36	0.719	1.41

* for *p* < 0.05. *B* = ordinal logistic regression coefficient; *SE* = standard error; *OR* = odds ratio. Positive coefficients indicate higher odds of being classified into a higher category of preference for AI-based scoring and feedback.

**Table 7:** Themes in AI scoring and feedback reflections.

Theme	Description	*n*
AI as a formative learning aid	Preference for AI as a tool for learning, feedback, or improvement	32
Human authority	Preference for human judgment, nuance, emotional understanding, or final control	17
Context-dependent preference	Acceptance depends on the assignment type, stakes, creativity, or discipline	9
Skepticism toward educational impact	Concerns about harm to learning, fairness, bias, inconsistency, or critical thinking	18

Note: *n* refers to the number of coded references (responses may be assigned up to two themes per participant).

**Table 8:** Summary of sentiment scores for each topic.

Topic	Positive Words	Negative Words	Sentiment Score
Detailed feedback and examples	38	8	30
Usefulness and clarity of feedback	75	4	71
Accuracy of scoring and feedback	73	3	70

## Data Availability

The data presented in this study are available on request from the corresponding author due to ethical considerations.

## Abbreviations

The following abbreviations are used in this manuscript:

AI	Artificial Intelligence
LDA	Latent Dirichlet Allocation

## Appendix A. Background & AI Familiarity

1. What is your age? ______
2. What is your gender?
  Male | Female | Non-binary | Prefer not to say
3. What is your current academic level?
  First-year | Second-year | Third-year | Fourth-year or higher
4. What is your major or area of study? ______
5. What is your current GPA (Grade Point Average)?
  4.0 | 3.99–3.5 | 3.49–3.0 | 2.99–2.5 | 2.45–2.0 | 1.99 or below | Prefer not to disclose
6. How comfortable are you with technology in general?
  Very uncomfortable | Somewhat uncomfortable | Somewhat comfortable | Very comfortable
7. How familiar are you with artificial intelligence (AI)?
  Not familiar at all | Not very familiar | Somewhat familiar | Very familiar
8. How familiar are you with AI scoring in writing tasks?
  Not familiar at all | Not very familiar | Somewhat familiar | Very familiar
9. How familiar are you with AI-generated feedback in writing tasks?
  Not familiar at all | Not very familiar | Somewhat familiar | Very familiar
10. How often do you receive feedback on your assignments from human graders?
  Daily | Weekly | Monthly | Occasionally | Rarely/Never
11. How frequently do you use AI tools (e.g., ChatGPT, Grammarly)?
  Daily | Weekly | Monthly | Occasionally | Rarely/Never
12. For which of the following purposes do you use AI tools (e.g., ChatGPT, Grammarly)? (Select all that apply)
  ⬤ Enhancing learning and understanding
  ⬤ Summarizing course content or materials
  ⬤ Proofreading and correcting grammar, spelling, and punctuation
  ⬤ Generating ideas or brainstorming
  ⬤ Writing drafts or essays
  ⬤ Language translation
  ⬤ Other (please specify)

## Appendix B. Task 1

Below are three student responses to the question: “If your school could offer a new course, what would you want it to be? Explain your answer using specific details.”

Each response is accompanied by two scores and two feedback. For each response, please indicate which score and feedback you prefer.

Response 1: If my school can offer a new course I would want it to be Hairstyling. Hairstyling is a course for me to show my talent and be creative, when I do hair this is a way for me to express my feelings and also myself.

Score: 2/3 & Feedback: The response identifies a new course (Hairstyling) and provides a vague explanation (…to show my talent and be creative, when I do hair this is a way to express my feelings) for why the course was chosen. The reader needs to make the connection between the support provided and what it is intended to prove.

Score: 3/3 & Feedback: Response is developed with clear, specific, and relevant ideas and information. The response identifies a new course (Hairstyling) and provides a clear explanation using specific and relevant details.

Response 2: A new course I would offer to the school would be a course where you study a different country/province/territory/state each day. Then at the end of the week you pick one food from one of the places you studied that week and you eat the food in class.

Score: 2/3 & Feedback: Response is developed with vague ideas and information; it may contain some irrelevant ideas and information. The response identifies a new course (study different regions and eat food) but provides a vague explanation and details.

Score: 1/3 & Feedback: The response describes a course (study a different country…eat the food) but does not provide an explanation for why the course was chosen.

Response 3: If my school was going to offer a new course I would want it to be a cooking/baking class. I would hope it would be this because I love to cook and have a great interest in learning more recipes and cooking techniques. I also think this class would be helpful because cooking helps with math skills like measurement, unit conversion, and comprehension skills for understanding a recipe.

Score: 3/3 & Feedback: The response identifies a new course (cooking/baking) and provides specific and relevant details (helps with math skills like measurement, unit conversion, and comprehension skills for understanding a recipe) to clearly explain why the course was chosen.

Score: 3/3 & Feedback: Response is developed with clear, specific, and relevant ideas and information. The response identifies a new course (Cooking/Baking class) and provides a clear explanation using specific and relevant details.

## Appendix C. AI Scoring and Feedback Preferences

For each situation below, please select your preferred method of scoring and feedback for the type of assignment described.

⬤ Score and feedback by AI
⬤ Score by AI, feedback by a human
⬤ Score by a human, feedback by AI
⬤ Score and feedback by a human

1. Which scoring and feedback method do you prefer in general for academic writing assignments?
2. Which scoring and feedback method do you prefer for low-stakes assignments (e.g., practice essays, writing exercises)?
3. Which scoring and feedback method do you prefer for high-stakes assignments (e.g., final papers, term projects)?
4. Which scoring and feedback method do you prefer if the assignment involves creative or subjective writing (e.g., personal reflections)?
5. Which scoring and feedback method do you prefer if you have the option to appeal or request a second review afterward?

Select the option that best matches what you find most helpful or effective.

1. What tone do you prefer in AI-generated feedback?
  Encouraging | Neutral | Critical | A mix depending on the task
2. What length of AI feedback do you prefer?
  Concise—short, to-the-point suggestions
  Detailed—thorough explanations and examples
  A combination of both—key points plus some detailed guidance
  Depends on the assignment—concise for low-stakes, detailed for high-stakes
3. What format do you prefer for AI feedback?
  Bulleted points | Paragraph-style | Inline comments on the text | A mix of formats
4. When would you prefer to receive AI feedback in your writing process?
  After the first draft | After revisions | Before submission | At multiple stages
5. Do you prefer AI feedback to focus most on:
  Content and ideas | Grammar and mechanics | Organization and structure | Style and tone

## Appendix D. Scenarios

For each of the following scenarios, please select your preferred method of scoring and feedback for the assignment described.

⬤ Score and feedback by AI
⬤ Score by AI, feedback by a human
⬤ Score by a human, feedback by AI
⬤ Score and feedback by a human

1. Scenario 1: You are submitting a final research paper that counts for 40% of your overall course grade.
2. Scenario 2: You submit a draft of an essay to receive feedback before the final version is due. The goal is to improve your work through revision.
3. Scenario 3: You are asked to write a personal reflection that includes creative elements.
4. Scenario 4: You are submitting a paper and know you will have the option to request a second review or appeal the score if needed.
5. Scenario 5: You are submitting an essay on a highly technical topic where the accuracy of the content is critical.
6. Scenario 6: Your instructor assigns a short, ungraded writing task to help you practice structuring arguments. You are told it will not impact your final grade.
7. Scenario 7: You are submitting a final research paper that counts for 10% of your course grade.
8. Scenario 8: You are nearing a submission deadline in one week. Your instructor offers an option to have your draft scored and receive feedback from an AI system within 24 h, allowing you to revise before the final submission. Alternatively, you can wait for human scoring and feedback, which will take 6 days.
9. Scenario 9: You are asked to write a personal narrative about a difficult life experience for a psychology course.
10. Scenario 10: You are a non-native English speaker submitting an argumentative essay for a writing-intensive course. You are concerned about misinterpretation of your phrasing or being penalized for grammar issues.

## Appendix E. AI Perceptions

Please indicate your level of agreement with the following statements about AI scoring and feedback. Strongly Disagree–Disagree–Agree–Strongly Agree.

### Appendix E.1. AI Scoring

1. AI scoring is objective.
2. AI scoring is consistent.
3. AI scoring is transparent and explainable.
4. AI can fairly grade diverse writing styles and voices.
5. AI can accurately grade grammar and mechanics in writing.
6. AI can accurately grade the organization and clarity of ideas in writing.
7. AI can accurately grade complex or creative writing.
8. AI assigns similar scores to similar quality work.
9. AI grading might overlook important aspects of my writing. (reverse-coded)
10. AI grading might struggle to interpret the tone or emotion in my writing. (reverse-coded)

### Appendix E.2. AI Feedback

1. AI feedback is easy to understand.
2. AI feedback gives me clear next steps for improvement.
3. AI feedback clearly identifies what I did well in my writing.
4. AI feedback clearly identifies what I need to improve.
5. AI feedback avoids vague or generic comments.
6. AI feedback is relevant to the content of my writing.
7. AI feedback helps me become more independent as a writer.
8. AI feedback is too general to be useful. (reverse-coded)
9. AI feedback is repetitive or redundant. (reverse-coded)
10. AI feedback sometimes contradicts itself. (reverse-coded)

## Appendix F. AI Preferences

Please indicate your level of agreement with the following statements about AI scoring and feedback. Strongly Disagree–Disagree–Agree–Strongly Agree.

### Appendix F.1. Scoring Process Preferences

1. I prefer to see a breakdown of how the AI grades each part of my writing.
2. I prefer to receive a confidence level or accuracy rating along with the AI scoring and feedback.
3. I prefer to have the choice to opt in or opt out of AI scoring and feedback.
4. I prefer AI if it includes a justification based on a clear rubric.
5. I prefer AI if its decision-making process is fully transparent.
6. I prefer AI to only offer feedback, not assign scores.
7. I prefer AI feedback to highlight both strengths and weaknesses in my writing.
8. I prefer AI feedback to reference specific examples from my writing.
9. I prefer AI feedback to include links or resources for further learning.
10. I prefer AI to allow me to ask follow-up questions about its score and feedback.

### Appendix F.2. AI vs. Human Comparison

1. I prefer AI over a human grader for low-stakes assignments (e.g., practice essays).
2. I prefer AI over a human grader for high-stakes assignments (e.g., final papers).
3. I prefer AI scoring and feedback to always be reviewed by a human.
4. I prefer to receive AI-generated feedback but human-assigned scores.
5. I prefer that AI be used only to assist human graders, not replace them.
6. I prefer my score to be equally based on AI and human evaluation.
7. I prefer AI for fast self-assessment before submission, instead of waiting for a human grader.
8. I prefer AI involvement in final grading, rather than relying entirely on a human grader.
9. I prefer to see a side-by-side comparison of AI and human scoring and feedback.
10. I prefer AI scoring and feedback first, then human, to maximize both speed and insight.

## Appendix G. Task 2

Please complete this short activity:

⬤ Copy the question, scoring rubric, and short student response below.
⬤ Paste them into ChatGPT and ask it to: “Provide a score and feedback for this response based on the rubric”.
⬤ Review the AI’s score and feedback. Once done, return to the survey and answer the question below:
⬤ Please copy and paste the score and feedback generated by ChatGPT. Then, reflect on your impressions: To what extent did you find the score accurate? Was the feedback clear and constructive? In your view, how useful (or not) might AI-generated scoring and feedback be for students?

Copy the question, scoring rubric, and short student response below:

In your opinion, should a university require students to take at least one class in AI ethics, regardless of their major? Explain your reasoning.

Scoring Rubric (0–4 points)

⬤ 4 points—Clear, well-developed argument with specific examples; logical structure; minimal grammar errors.
⬤ 3 points—Good argument but may lack depth or specific examples; mostly clear; few grammar errors.
⬤ 2 points—Argument is underdeveloped or unclear in places; limited examples; some grammar errors.
⬤ 1 point—Weak or unclear argument; few or no examples; multiple grammar issues.
⬤ 0 points—Off-topic, incomprehensible, or no answer provided.

Student Response: I think AI ethics classes could be useful for students because they can teach important rules and guidelines for using AI responsibly. However, I don’t think every student needs to take one since some majors may not use AI much. Still, having some knowledge of AI ethics could help students make better decisions and avoid problems in their future careers.
